# Effect of Endometrium Thickness on Clinical Outcomes in Luteal Phase Short-Acting GnRH-a Long Protocol and GnRH-Ant Protocol

**DOI:** 10.3389/fendo.2021.578783

**Published:** 2021-05-17

**Authors:** Jie Zhang, Yi-Fei Sun, Yue-Ming Xu, Bao-jun Shi, Yan Han, Zhuo-Ye Luo, Zhi-Ming Zhao, Gui-Min Hao, Bu-Lang Gao

**Affiliations:** Department of Reproductive Medicine, The Second Hospital of Hebei Medical University, Shijiazhuang, China

**Keywords:** gonadotropin-releasing hormone agonist, gonadotropin releasing hormone antagonist, pregnancy outcome, endometrial thickness, clinical pregnancy

## Abstract

**Objective:**

To investigate the factors that influence luteal phase short-acting gonadotropin-releasing hormone agonist (GnRH-a) long protocol and GnRH-antagonist (GnRH-ant) protocol on pregnancy outcome and quantify the influence. About the statistical analysis, it is not correct for the number of gravidities.

**Methods:**

Infertile patients (n = 4,631) with fresh *in*-*vitro* fertilization/intracytoplasmic sperm injection (IVF/ICSI) and embryo transfer were divided into GnRH-a long protocol (n =3,104) and GnRH-ant (n =1,527) protocol groups and subgroups G1 (EMT ≤7mm), G2 (7 mm <EMT ≤10 mm), and G3 (EMT >10 mm) according to EMT on the trigger day. The data were analyzed.

**Results:**

The GnRH-ant and the GnRH-a long protocols had comparable clinical outcomes in the clinical pregnancy, live birth, and miscarriage rate after propensity score matching. In the medium endometrial thickness of 7–10 mm, the clinical pregnancy rate (61.81 *vs* 55.58%, P < 0.05) and miscarriage rate (19.43 *vs* 12.83%, P < 0.05) of the GnRH-ant regime were significantly higher than those of the GnRH-a regime. The EMT threshold for clinical pregnancy rate in the GnRH-ant group was 12 mm, with the maximal clinical pregnancy rate of less than 75% and the maximal live birth rate of 70%. In the GnRH-a long protocol, the optimal range of EMT was >10 mm for the clinical pregnancy rate and >9.5 mm for the live birth rate for favorable clinical outcomes, and the clinical pregnancy and live birth rates increased linearly with increase of EMT. In the GnRH-ant protocol, the EMT thresholds were 9–6 mm for the clinical pregnancy rate and 9.5–15.5 mm for the live birth rate.

**Conclusions:**

The GnRH-ant protocol has better clinical pregnancy outcomes when the endometrial thickness is in the medium thickness range of 7–10 mm. The optimal threshold interval for better clinical pregnancy outcomes of the GnRH-ant protocol is significantly narrower than that of the GnRH-a protocol. When the endometrial thickness exceeds 12 mm, the clinical pregnancy rate and live birth rate of the GnRH-ant protocol show a significant downward trend, probably indicating some negative effects of GnRH-ant on the endometrial receptivity to cause a decrease of the clinical pregnancy rate and live birth rate if the endometrial thickness exceeds 12 mm.

## Introduction

In the controlled ovarian stimulation (COS) process, the gonadotropin-releasing hormone antagonist (GnRH-ant) protocol plays an increasingly important role compared with the classic protocol, the luteal phase short-acting gonadotropin-releasing hormone agonist (GnRH-a) long protocol because of the advantages of the GnRH-ant protocol which are more in line with the physiological processes. These advantages include a low dose of medication, high compliance of the patients, a low risk of early COS failure, quick interaction with the body’s receptors, and lack of low estrogen symptoms (1). The impact of GnRH-ant protocol on the clinical pregnancy rate is controversial. Early research showed that the GnRH-ant protocol had a lower pregnancy rate than the GnRH-a long protocol (2). But a recent meta-analysis showed that in terms of live birth rate, there was no statistically significant difference between the GnRH-ant and GnRH-a long protocols (3). After comparing the clinical outcomes in GnRH-ant and GnRH-a treatment for ovulation and the pregnancy outcomes of subsequent frozen–thawed embryo transfer, Bahceci et al. (4) found that the implantation rate in the fresh embryo transfer cycle was lower in the GnRH-ant than that in the GnRH-a long protocol group, but the implantation rate and clinical pregnancy rate did not decrease in subsequent frozen–thawed cycles. This may indicate that the use of GnRH-ant may adversely affect the endometrial receptivity but does not affect oocyte quality and embryo development. In assisted reproductive technology (ART), the commonly used indexes of endometrial receptivity include (5, 6) ultrasound imaging and morphological signs: thickness and type of endometrium, uterine arteries, and sub-endometrial blood flow; protein markers: leukemia inhibitory factor, matrix metalloproteinases, cell adhesion molecules, osteopontin, and Wnt signal transduction system; genetic markers: homeobox gene, HOX gene, and gene chip. Endometrial thickness (EMT) and morphology have become the most commonly used indicators for clinical assessment of endometrial receptivity because of the non-invasiveness, convenience, and economy in measuring the endometrium. Researchers have found that the clinical pregnancy rate of women with EMT ≤7 mm is 23.3%, which is significantly lower than that (48.1%) of women with the EMT >7 mm (7). Hence, thin endometrium is often defined as less than 7 mm during the late follicular phase or after ovulation (8). A study about the fresh embryo transfer cycle found that when the EMT threshold was 10 mm or more, the live births were maximized, while the pregnancy losses were minimized (9). However, the optimal EMT is unknown for achieving the best clinical pregnancy outcomes. In this retrospective cohort study, patients who had undergone fresh *in-vitr*o fertilization (IVF)/intracytoplasmic sperm injection (ICSI) embryo transfer at our hospital were investigated, and the clinical pregnancy outcomes and the relationship between EMT on the trigger day and clinical outcomes in GnRH-a long and GnRH-ant protocols were analyzed to find the optimal EMT.

## Method

### Subjects

**Figure 1:**
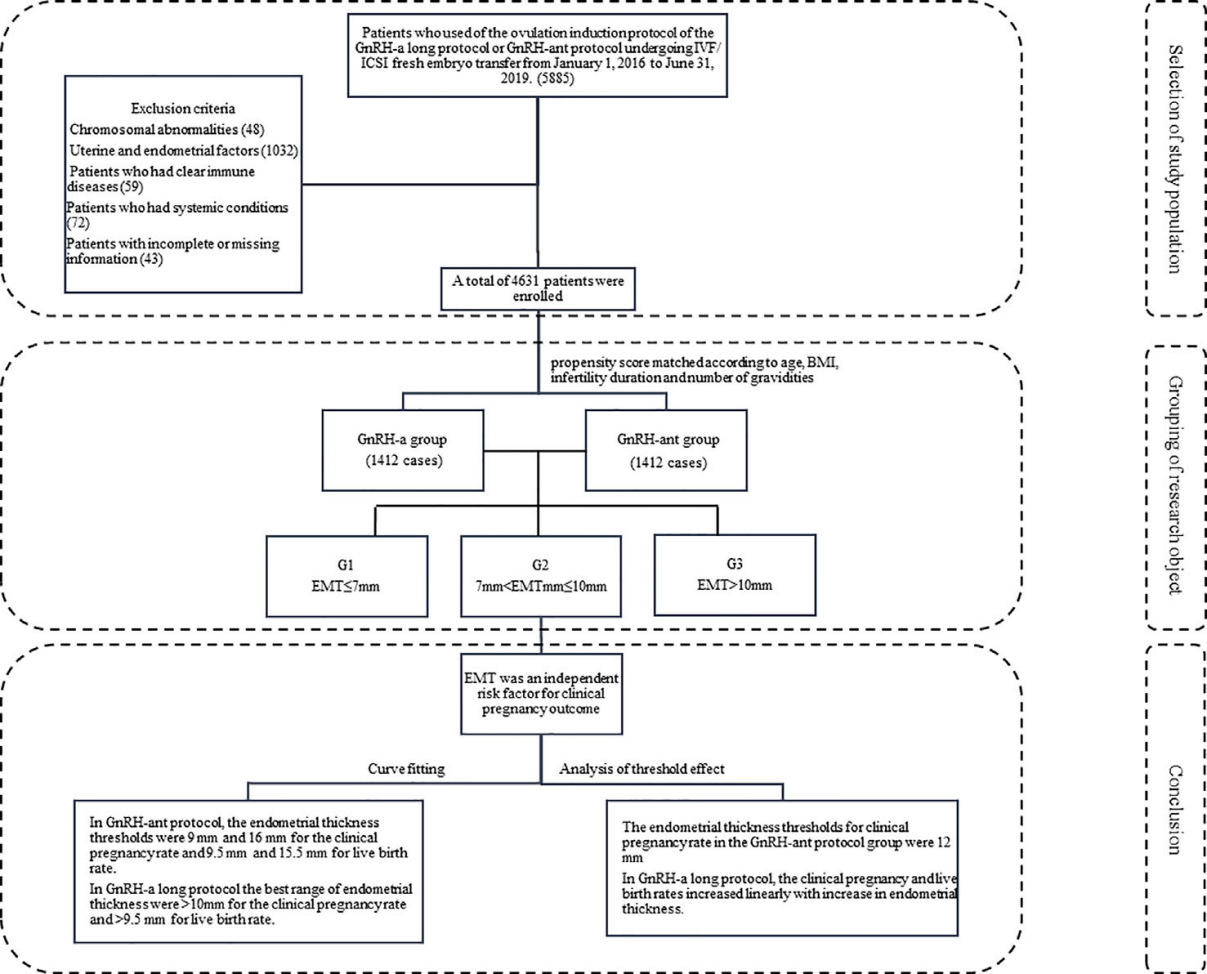
Flow diagram of selection process and conclusion.

This retrospective study was approved by the ethics committee of the Second Hospital of Hebei Medical University, and all patients had given their signed informed consent to participate. All methods were performed in accordance with the relevant guidelines and regulations. Patients who underwent fresh IVF/ICSI embryo transfer from January 1, 2016 to June 31, 2019 at our hospital were enrolled. The inclusion criteria were use of the ovulation induction protocol of the GnRH-a or GnRH-ant, normal chromosome karyotype, application of fresh embryo transfer, and even endometrial echo. The exclusion criteria were chromosomal abnormalities of either husband or wife and uterine or endometrial conditions affecting the outcome of pregnancy, such as uterine malformations, uterine fibroids, adenomyoma, endometrial polyps, intrauterine adhesions, history of endometrial tuberculosis, and hydrosalpinx return to the uterine cavity. Patients who took glucocorticoids or immunosuppressants during the treatment or who had infectious diseases including pneumonia, pelvic inflammatory disease, and urinary tract infection were excluded. Patients with systematic diseases like diabetes mellitus or thyroid disease were also excluded (**Figure 1**).

### Treatment Protocol

#### Controlled Ovarian Hyperstimulation Protocol

In the GnRH-a long protocol, patients were treated with daily injection of 0.1 mg or 0.05 mg triptorelin acetate (Triptorelin, Ferring GmbH, Kiel, Germany, specifications: 1 ml; 0.1 mg) beginning in the middle of the luteal phase of the previous menstrual cycle. The entire injection period lasted 14–16 days. The serum hormone levels (follicle-stimulating hormone (FSH), luteinizing hormone (LH), estradiol (E_2_), progesterone, and B-ultrasound were checked on the 3rd to 5th day of the menstruation cycle, and then, gonadotropin (Gn) (Recombinant Human Follitropin Alfa for Injection, MerckSeronoS.p.A, Geneva, Switzerland, Specification: 5.5 μg or 75 IU) was used after the down-regulation standard was reached (FSH ≤5 mIU/ml, LH ≤5 mIU/ml, E_2_ ≤50 pg/ml, progesterone ≤1.5 ng/ml, and EMT ≤5 mm). The initial dose of Gn was 125–375 IU per day, depending on the patient’s age, body mass index (BMI), basal follicle number, basal serum FSH (bFSH), and anti-Mullerian hormone (AMH) level. The dose of Gn was adjusted according to the growth of the follicle and the hormone results.

#### GnRH-ant Protocol

Gn (Recombinant Human Follitropin Alfa for Injection, MerckSeronoS.p.A, Geneva, Switzerland, Specification: 5.5 μg or 75 IU) was administered on the second or third day of the menstrual cycle, with the initial dose of Gn being determined on the female age, BMI, basal follicle number, bFSH, and ovarian reserve. After Gn was administered for 4–5 days, the dose was adjusted according to the patient’s reactivity to the drug. When the dominant follicle diameter was ≥14 mm, E_2_ ≥400 pg/ml, or LH ≥10 mIU/ml, subcutaneous injection of GnRH-ant (Cetrorelix acetate powder for injection, MerckSeronoS.p.A, Geneva, Switzerland, Specification:0.25 mg) was started daily at 0.125–0.5 mg.

Human chorionic gonadotropin (HCG) (Chorionic Gonadotrophin for Injection, Livzon Pharmaceutical Group Inc., Zhuhai, China, specification: 2,000 IU) of 6,000–12,000 IU was injected when the largest follicle diameter was bigger than 18 mm or the diameter of at least three follicles was bigger than 17 mm. The dose of HCG was determined according to the BMI of the patient and the serum E_2_ level on the trigger day. The oocytes were retrieved under ultrasound guidance 36–38 h after injection of HCG. The AlokaSSD-Alpha 7 transvaginal ultrasound system with the 6.67 MHz 2D probe (Hitachi Medical, Tokyo, Japan) was used to measure the thickness of the endometrium on the trigger day. When measuring the thickness of the endometrium, the sagittal plane of the uterus was chosen to display the cervical ostium and the uterine floor simultaneously, and the maximal distance between the junction of the myometrium and the endometrium on both sides was measured. The measurement was performed by experienced sonographers; the unit of measurement was millimeter (mm), and the highest accuracy was 0.1 mm. The endometrial echo type was measured at the same time. The endometrial classification is divided into three types: A, B, and C. Type A is of three-line or multi-layer endometrium, which is characterized by the outer and middle strong echo and the inner hypoechoic or non-echoic area, with obvious midline echo of the uterine cavity; Type B is of a uniform, moderate-intensity echo, with intermittent midline echo of the official cavity; Type C is of a homogeneous and strong echo, with no midline echo of the uterine cavity.

The following criteria were for cancellation of embryo transfer. In order to prevent ovarian hyperstimulation syndrome (OHSS), embryos should not be transferred for patients with ≥15 oocytes, a large amount of ascites found on ultrasound examination, or symptoms such as abdominal pain, bloating, nausea, and vomiting. Patients with serum progesterone level ≥2.0 ng/ml on the trigger day or poor endometrial state including ultrasound prompting strong endometrial echo, uneven endometrial echo, poor endometrial morphology, and uterine effusion were also excluded from embryo transfer. *In-vitro* fertilization was performed after the oocyte was taken. Patients without transferrable embryos had to abandon the transfer. Patients with suspected hydrosalpinx on one or both sides or a dark area of extraovarian fluid or vaginal discharge on multiple ultrasound examinations were excluded from embryo transfer. Other factors like fever on the day of transplantation, inability to visit for personal reasons, or abnormal thyroid function also precluded the patient from receiving embryo transfer. For patients with less than 7 mm of EMT but normal endometrial morphology, a low pregnancy rate might result, and it was up to the patient to receive embryo transfer after being fully informed.

#### Outcomes and Definition of Indicators

The method of fertilization was IVF or ICSI, according to the male semen. Seventy-two hours after oocyte retrieval, transplantation was decided based on embryos grading, hormone levels, and endometrial condition. The data of patients in two groups were analyzed including female age, infertility duration (month, mo), number of gravidities, BMI, bFSH, basic serum luteinizing hormone level (bLH), basic serum luteinizing hormone level (bAMH), basic serum progesterone level (bP), basic serum Estradiol level (bE_2_), basic serum Testosterone level (bT), total dose of Gn, total dose of GnRH-a, total dose of GnRH-ant, EMT on the trigger day, number of embryos transferred, biochemical pregnancy, and clinical pregnancy outcomes (clinical pregnancy rate, live birth rate, and miscarriage rate). In the process of transplantation, the “Management Measures of Human Assisted Reproductive Technology” issued by the Ministry of Health of China was strictly abided by: women under 35 years cannot be transferred more than two embryos for the first time, but three embryos can be transferred for women at the second time. Biochemical pregnancy was defined as serum HCG greater than 25 mIU/ml 12–14 days after transfer. Gynecological ultrasound examination was performed 30–35 days after transfer, and the presence of a gestational sac was considered as clinical pregnancy, with the clinical pregnancy rate = (number of clinical pregnancy cycles/number of all transfer cycles) × 100%, and live birth rate = (number of live birth cycles/number of all transfer cycles) × 100%. If the pregnancy was less than 28 weeks and the fetus weighed less than 1,000 g when the pregnancy was terminated, it was defined as abortion, with the abortion rate = (number of abortion cycles/number of all clinical pregnancy cycles) × 100%.

### Statistical Analysis

All statistical analyses were performed with the SPSS 25.0 statistical software (IBM, Chicago, IL, USA) or the statistical packages R (The R Foundation; version 3.4.3) or Empower (R) (X&Y solutions, Boston, MA, USA). Data with normal distribution were expressed as mean ± standard deviation (SD), and data with non-normal distribution were expressed as median (quartile range). In normal distribution, two independent samples’ test was used to compare means between two groups, and one-way ANOVA analysis of variance was used to compare means among multiple groups. In non-normal distribution, non-parametric test (Mann–Whitney U-test) was used to compare the means. The comparison of counted data was performed with the Chi-square test or Fisher exact probability method. Various factors affecting clinical outcomes were identified by univariate logistic regression analysis, and multivariate logistic regression analysis was performed to adjust confounding factors for studying the effect of EMT on clinical outcomes. After adjusting confounding factors, the smooth curve fitting was used to observe the relationship between EMT and pregnancy outcomes. A piecewise regression model was used to analyze the threshold effect of EMT and clinical outcomes. The smooth curve fitting and threshold effect value were combined to quantify the effect of EMT on clinical pregnancy outcomes in different ovulation protocols. The statistical significance was set at P <0.05.

## Results

### Subjects

**Figure 2:**
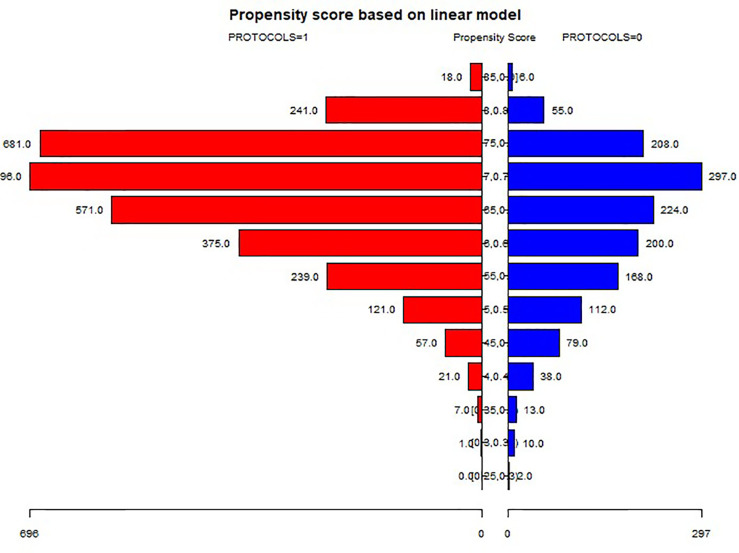
Propensity score matching of the two protocols.

A total of 4m631 patients met the inclusion criteria and were divided into the GnRH-a long (n = 3104) and GnRH-ant (n = 1527) protocol groups according to the ovulation induction protocol. 2,824 patients were screened after 1:1 propensity score matching according to age, BMI, infertility duration, and number of gravidities (**Figure 2**) and were assigned to two treatment groups with 1,412 patients in each group. Three subgroups were established according to the EMT on the trigger day: G1 (EMT ≤7 mm), G2 (7 mm <EMT ≤10 mm), and G3 (EMT >10 mm). Comparison between different protocol groups was performed. The results of normality test showed that the data involved in this article were all non-normally distributed and tested accordingly.

A significant (P < 0.05) difference existed in the female age, infertility duration, number of gravidities, BMI, bFSH, bE_2_, bP, EMT on the trigger day, number of embryos transferred, and miscarriage rate between the GnRH-a long and GnRH-ant groups (**Table 1**). After propensity score matching with age, infertility duration, number of gravidities, and BMI, the significant differences between two groups were in bFSH, bE_2_, bAMH, total dose of Gn, EMT on the trigger day, and number of embryos transferred (**Table 2**). There was no significant difference (P > 0.05) in bP, bLH, bT, endometrial echo type, clinical pregnancy rate, live birth rate, and miscarriage rate between the two groups.

**Table 1:** Clinical data and pregnancy outcomes in different protocols.

Variables	GnRH-a (n = 3,104)	GnRH-ant (n = 1,527)	P
Age (y)	30.00 (6.00)	31.00 (8.00)	<0.05
Infertility duration (mo)	36.00 (36.00	38.00 (48.00)	<0.05
Number of gravidities	2.00 (1.00)	1.00 (1.00)	<0.05
BMI (kg/m(^2^))	23.08 (3.36)	23.68 (3.59)	<0.05
bFSH (mIU/mL)	7.13 (2.42)	7.62 (3.34)	<0.05
bE_2_ (pg/mL)	37.00 (28.00)	39.00 (28.48)	<0.05
bP (ng/mL)	0.66 (0.55)	0.64 (0.51)	<0.05
bLH (mIU/mL)	4.11 (2.44)	4.05 (2.72)	>0.05
bT (ng/mL)	0.42 (0.25)	0.41 (0.26)	>0.05
bAMH (ng/mL)	2.71 (2.12)	1.81 (2.25)	>0.05
Total dose of Gn (IU)	2400 (975)	2400 (1050)	>0.05
EMT (mm)	10.00 (2.00)	11.00 (2.00)	<0.05
Endometrial echo type			
A	63.0% (1955/3104)	65.0% (993/1527)	>0.05
B	35.4% (1099/3104)	32.6% (498/1527)	>0.05
C	1.6% (50/3104)	2.4% (36/1527)	>0.05
Number of embryos transferred			
1	5.3% (165/3104)	11.4% (173/1527)	<0.05
2	93.2% (2892/3104)	83.4% (1274/1527)	<0.05
3	1.5% (47/3104)	5.2% (80/1527)	<0.05
Clinical pregnancy rate	64.7% (2008/3104)	66.9% (1022/1527)	>0.05
Live birth rate	54.9% (1705/3104)	53.8% (821/1527)	>0.05
Miscarriage rate	13.0% (262/2008)	15.9% (163/1022)	<0.05

GnRH-a, gonadotropin-releasing hormone agonist; GnRH-ant, gonadotropin-releasing hormone antagonist; mo, month; BMI, body mass index; bFSH, basal follicle-stimulating hormone; bE, baseline estradiol; bP, baseline progesterone; bLH, baseline luteinizing hormone; bT, baseline testosterone; bAMH, baseline anti-Mullerian hormone; EMT, Endometrial thickness.

**Table 2:** Clinical data and pregnancy outcomes in different protocols after Propensity score matching.

Variables	GnRH-a (n = 1,412)	GnRH-ant (n = 1,412)	P
Age (y)	31.00 (7.00)	31.00 (7.00)	>0.05
Infertility duration (mo)	36.00 (48.00)	36.00 (45.00)	>0.05
Number of gravidities	1.00 (1.00)	1.00 (1.00)	>0.05
BMI (kg/m^2^)	23.23 (4.71)	23.23 (4.59)	>0.05
bFSH (mIU/ml)	7.04 (2.34)	7.62 (3.36)	<0.05
bE_2_ (pg/ml)	37.00 (29.00)	39.00 (28.31)	<0.05
bP (ng/ml)	0.64 (0.54)	0.64 (0.50)	>0.05
bLH (mIU/ml)	3.97 (2.45)	4.10 (2.75)	>0.05
bT (ng/ml)	0.40 (0.27)	0.42 (0.26)	>0.05
bAMH (ng/ml)	2.65 (2.32)	1.80 (2.23)	<0.05
Total dose of Gn (IU)	2400 (975)	2400 (1050)	<0.05
EMT (mm)	11.00 (2.00)	10.00 (2.00)	<0.05
Endometrial echo type			
A	63.0% (889/1412)	65.8% (929/1412)	>0.05
B	35.4% (500/1412)	32.5% (459/1412)	>0.05
C	1.6% (23/1412)	1.7% (24/1412)	>0.05
Number of embryos transferred			
1	7.58% (107/1412)	13.03% (184/1412)	<0.05
2	90.93% (1284/1412)	81.59% (1152/1412)	<0.05
3	1.49% (21/1412)	5.38% (76/1412)	<0.05
Clinical pregnancy rate	62.89% (888/1412)	65.23% (921/1412)	>0.05
Live birth rate	54.11% (764/1412)	54.25% (766/1412)	>0.05
Miscarriage rate	13.96% (124/888)	16.83% (155/921)	>0.05

GnRH-a, gonadotropin-releasing hormone agonist; GnRH-ant, gonadotropin-releasing hormone antagonist; mo, month; BMI, body mass index; bFSH, basal follicle-stimulating hormone; bE_2_, baseline estradiol; bP, baseline progesterone; bLH, baseline luteinizing hormone; bT, baseline testosterone; bAMH, baseline anti-Mullerian hormone; EMT, Endometrial thickness.

### Comparison Between Different Subgroups

Patients in the GnRH-a long protocol group were divided into three subgroups according to the EMT on the trigger day (**Table 3**), and a significant (P < 0.05) difference existed in female age, BMI, number of gravidities, bFSH, clinical pregnancy rate, and live birth rate among three subgroups. Subgroup G3 had a significantly (P < 0.05) greater clinical pregnancy rate and a live birth rate than those in the other two subgroups. No significant (P > 0.05) difference was detected in the miscarriage rate among the three groups.

**Table 3:** Clinical data and pregnancy outcomes in subgroups of GnRH-a long protocol.

Variables	G1	G2	G3	P
N	36	547	829	
Age (y)	37.00 (9.50)	31.00 (6.00)	31.00 (6.00)	<0.05
Infertility duration (mo)	48.00 (25.50)	36.00 (38.50)	36.00 (48.00)	>0.05
BMI (kg/m^2^)	23.10 (5.01)	22.90 (4.70)	23.40 (3.74)	<0.05
Number of gravidities	1.00 (2.00)	1.00 (2.00)	1.00 (1.00)	<0.05
bFSH (mIU/ml)	7.66 (1.62)	7.07 (2.33)	7.01 (2.36)	>0.05
bE_2_ (pg/ml)	42.50 (28.50)	37.00 (27.30)	36.00 (28.50)	>0.05
bP (ng/ml)	0.51 (0.37)	0.68 (0.53)	0.61 (0.55)	>0.05
bLH (mIU/ml)	3.80 (1.22)	4.02 (2.44)	3,89 (2.49)	>0.05
bT (ng/ml)	0.37 (0.31)	0.41 (0.26)	0.40 (0.27)	>0.05
bAMH (ng/ml)	4.53 (3.29)	2.33 (2.10)	2.74 (2.29)	>0.05
Total dose of Gn(IU)	2362.50 (375.00)	2475.00 (975.00)	2475.00 (975.00)	>0.05
Total dose of GnRH-a(mg)	0.50 (0.13)	0.50 (0.15)	0.50 (0.11)	>0.05
Number of embryos transferred				
1	2.78% (1/36)	7.68% (42/547)	7.72% (64/829)	>0.05
2	97.22% (35/36)	90.49% (495/547)	90.95% (754/829)	>0.05
3	0% (0/36)	1.83% (10/547)	1.33% (11/829)	>0.05
Endometrial echo type				
A	55.6% (20/36)	63.4% (347/547)	63.0% (522/829)	>0.05
B	41.7% (15/36)	36.2% (198/547)	34.6% (287/829)	>0.05
C	2.8% (1/36)	0.4% (2/547)	2.4% (20/829)	>0.05
Clinical pregnancy rate	50.00% (18/36)_a_	55.58% (304/547)_a_	68.28% (566/829)_b_	<0.05
Live birth rate	47.06% (8/36)_a_	48.45% (265/547)_a_	58.26% (483/829)_b_	<0.05
Miscarriage rate	11.11% (2/18)	12.83% (39/304)	14.66% (83/566)	>0.05

GnRH-a, gonadotropin-releasing hormone agonist; mo, month; BMI, body mass index; bFSH, basal follicle-stimulating hormone; bE_2_, baseline estradiol; bP, baseline progesterone; bLH, baseline luteinizing hormone; bT, baseline testosterone; bAMH, baseline anti-Mullerian hormone. Identical subscript letters indicate no significant difference while different subscript letters significant (P < 0.05) difference. Group G1, endometrial thickness ≤7.0 mm; Group G2, endometrial thickness belongs to 7.0–10.0 mm; Group G3, endometrial thickness >10.0 mm.

In the GnRH-ant group (**Table 4**), there was a significant (P < 0.05) difference in age, number of gravidities, bP, bT, bAMH, total dose of Gn, total dose of GnRH-ant, clinical pregnancy rate, and live birth rate among the three subgroups. Subgroup G3 also had a significantly (P < 0.05) higher clinical pregnancy rate and a live birth rate than those in the other groups. There was no significant (P > 0.05) difference in the miscarriage rate among three subgroups.

**Table 4:** Clinical data and pregnancy outcomes in subgroups of GnRH-ant protocol.

Variables	G1	G2	G3	P
N	76	741	595	
Age (y)	31.00 (7.00)	32.00 (7.00)	31.00 (7.00)	<0.05
Infertility duration (mo)	36.00 (48.00)	36.00 (42.00)	41.50 (48.00)	>0.05
BMI (kg/m^2^)	23.59 (4.28)	23.05 (4.59)	23.40 (4.56)	>0.05
Number of gravidities	1.00 (2.00)	1.00 (2.00)	0.00 (1.00)	<0.05
bFSH (mIU/ml)	7.80 (4.32)	7.67 (3.52)	7.56 (3.14)	>0.05
bE_2_ (pg/ml)	45.50 (28.25)	40.00 (29.00)	38.00 (29.00)	>0.05
bP (ng/ml)	0.65 (0.71)	0.68 (0.53)	0.61 (0.48)	<0.05
bLH (mIU/ml)	3.90 (2.23)	4.05 (2.74)	4.21 (2.81)	>0.05
bT (ng/ml)	0.37 (0.18)	0.43 (0.13)	0.40 (0.27)	<0.05
bAMH (ng/ml)	0.96 (0.71)	1.52 (1.64)	2.20 (3.07)	<0.05
Total dose of Gn(IU)	2100.00 (675.00)	2400.00 (975.00)	2400.00 (1125.00)	<0.05
Total dose of GnRH-ant(mg)	0.50 (0.50)	0.50 (0.63)	0.63 (0.62)	<0.05
Number of embryos transferred				
1	26.32% (20/76)	12.96% (96/741)	11.43% (68/595)	>0.05
2	71.05% (54/76)	81.11% (601/741)	83.53% (497/595)	>0.05
3	2.63% (2/76)	5.94% (44/741)	5.04% (30/595)	>0.05
Endometrial echo type				
A	60.5% (46/76)	67.7% (502/741)	64.0% (381/595)	>0.05
B	38.2% (29/76)	30.5% (226/741)	34.3% (204/595)	>0.05
C	1.3% (1/76)	1.8% (13/741)	1.7% (10/595)	>0.05
Clinical pregnancy rate	56.58% (43/76) _a_	61.81% (458/741) _a_	70.59% (420/595) _b_	<0.05
Live birth rate	44.74% (34/76) _a_	49.80% (369/741) _a_	61.01% (363/595) _b_	<0.05
Miscarriage rate	20.93% (9/43)	19.43% (89/458)	13.57% (57/420)	>0.05

GnRH-ant, gonadotropin-releasing hormone antagonist; mo, month; BMI, body mass index; bFSH, basal follicle-stimulating hormone; bE_2_, baseline estradiol; bP, baseline progesterone; bLH, baseline luteinizing hormone; bT, baseline testosterone; bAMH, baseline anti-Mullerian hormone. Identical subscript letters indicate no significant difference while different subscript letters significant (P < 0.05) difference. Group G1, endometrial thickness ≤7.0 mm; Group G2, endometrial thickness belongs to 7.0–10.0 mm; Group G3, endometrial thickness >10.0 mm.

Even though there was no statistically significant (P > 0.05) difference in the overall pregnancy rate between two treatment options, the clinical pregnancy rate (61.81 *vs* 55.58%) and miscarriage rate (19.43 *vs* 12.83%) were significantly (P < 0.05) higher in the GnRH-ant regime than those in the GnRH-a regime in subgroup G2 with the medium thickness of EMT (**Table 5**).

**Table 5:** Comparison of pregnancy outcomes between different treatment schemes in different subgroups.

	G1			G2			G3		
	GnRH-a	GnRH-ant	P	GnRH-a	GnRH-ant	P	GnRH-a	GnRH-ant	P
Clinical pregnancy rate	50.00% (18/36)	56.58% (43/76)	>0.05	55.58% (304/547)	61.81% (458/741)	<0.05	68.28% (566/829)	70.59% (420/595)	>0.05
Live birth rate	47.06% (8/36)	44.74% (34/76)	>0.05	48.45% (265/547)	49.80% (369/741)	>0.05	58.26% (483/829)	61.01% (363/595)	>0.05
Miscarriage rate	11.11% (2/18)	20.93% (9/43)	>0.05	12.83% (39/304)	19.43% (89/458)	<0.05	14.66% (83/566)	13.57% (57/420)	>0.05

Group G1, endometrial thickness ≤7.0 mm; Group G2, endometrial thickness between 7.0 and 10.0 mm; Group G3, endometrial thickness >10.0 mm.

### Logistic Regression Analysis

Univariate logistic regression analysis showed that EMT on the trigger day was a significant (P < 0.05) factor affecting the clinical pregnancy and live birth rates rather than the miscarriage rate in both two groups, whereas endometrial echo type had no effect on clinical pregnancy outcome. The effect of EMT on clinical outcomes was analyzed using multivariate logistic regression analysis after adjustment of confounding factors. Age, BMI, number of gravidities, and number of embryos transferred were used as adjusted variables to analyze the relationship between EMT and pregnancy outcome in the GnRH-a group. Age, number of gravidities, bP, bT, bAMH, total dose of Gn, total dose of GnRH-ant, and number of embryos transferred were used as adjusted variables to analyze the relationship between EMT and pregnancy outcome in the GnRH-ant group. The results showed that after adjusting for confounding factors, EMT was an independent risk factor significantly (P < 0.05) affecting clinical pregnancy and live birth rates but with no significant (P > 0.05) effect on miscarriage (**Table 6**).

**Table 6:** Logistics regression analysis of endometrial thickness and clinical outcomes.

	GnRH-a				GnRH-ant			
	Non-adjusted		Adjusted		Non-adjusted		Adjusted	
	OR (95%CI)	P	OR (95%CI)	P	OR (95%CI)	P	OR (95%CI)	P
Clinical pregnancy rate	1.146 (1.083,1.212)	<0.05	1.144 (1.081,1.210)	<0.05	1.113 (1.050,1.180)	<0.05	1.106 (1.036,1.180)	<0.05
Live birth rate	1.17 (1.059,1.178)	<0.05	1.115 (1.056,1.176)	<0.05	1.113 (1.053,1.176)	<0.05	1.094 (1.029,1.163)	<0.05
Miscarriage rate	0.992 (0.903,1.091)	>0.05	0.996 (0.905,1.095)	>0.05	0.929 (0.847,1.018)	>0.05	0.955 (0.864,1.056)	>0.05

Age, BMI, number of gravidities and number of embryos transferred were used as adjusted variables to analyze the relationship between endometrial thickness and pregnancy outcome in the GnRH-a group; Age, number of gravidities, bP, bT, bAMH, total dose of Gn, total dose of GnRH-ant and number of embryos transferred were used as adjusted variables to analyze the relationship between endometrial thickness and pregnancy outcome in the GnRH-ant group.

### Curve Fitting

After adjustment of confounding factors such as age, BMI, infertility duration, and number of transferred embryos, the curve fitting analysis was performed.

**Figure 3:**
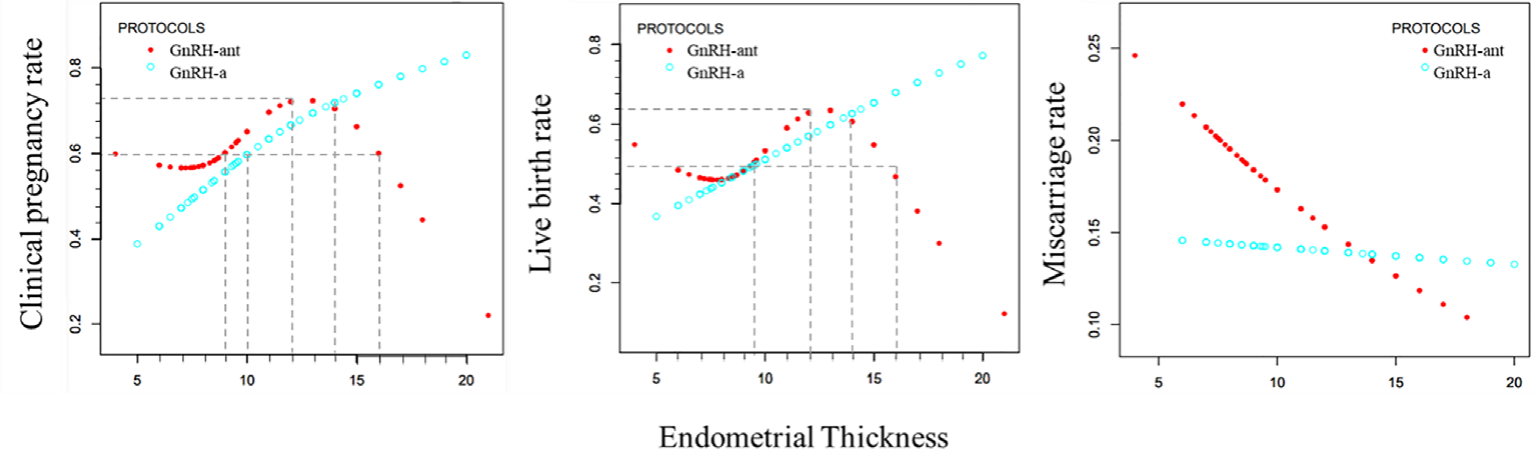
Curve fitting diagram of endometrial thickness and clinical outcome rate in GnRH-a long and GnRH-ant protocols.

In the GnRH-a group (**Figure 3**), the clinical pregnancy rate and live birth rate increased with increase of EMT, with the relationship fitting in a straight line. As the EMT increased, the miscarriage rate showed a slow downward trend (**Figure 3**).

In the GnRH-ant group (**Figure 3**), as the EMT increased, the clinical pregnancy rate and live birth rate first declined slowly, then significantly increased, and maintained at a high level before a sharp decline. The curve of miscarriage rate showed a state of decline.

The curve fitting analysis showed that in the GnRH-a group, the clinical pregnancy rate was ≥60% when the EMT was ≥10 mm, and the live birth rate was ≥50% when the EMT was ≥9.5 mm. In the GnRH-ant group, the clinical pregnancy rate was ≥60% when the EMT was between 9 and 16mm, and the live birth rate was ≥50% when the EMT was between 9.5 and 15.5 mm. In the GnRH-ant group, the optimal clinical pregnancy rate could be obtained when the EMT ranged 9–16 mm while the optimal live birth rate could be achieved when the EMT was between 9.5 and 15.5 mm.

Curve fitting analysis revealed that the GnRH-ant protocol had certain advantages when the EMT was less than 14 mm, but the GnRH-a long program had significant advantages when the EMT was greater than 14 mm. The maximal clinical pregnancy rate in the GnRH-ant group was 75%, and the maximal live birth rate was 70% when the EMT was 12 mm.

### Analysis of Threshold Effect

After adjustment of confounding factors, EMT was an important predictor of clinical outcomes. In the GnRH-a group, the clinical pregnancy rate increased by 14.4% and the live birth rate increased by 11.5% with every 1 mm increase in EMT (**Table 7**). In the GnRH-ant group, the EMT threshold for clinical pregnancy rate was 12 mm, the maximal clinical pregnancy rate was less than 75%, and the maximal live birth rate was 70%. When the EMT was thinner than 12 mm, the clinical pregnancy rate increased by 21.5% for every 1 mm increase in EMT, and the live birth rate increased by 19.6% with every 1 mm increase in EMT. When the EMT was thicker than 12 mm, the clinical pregnancy rate decreased by 17.5% for every 1 mm increase in EMT and the live birth rate decreased by 16% with every 1 mm increase in EMT (**Table 7**). Curvilinear relationship analysis demonstrated no significant (P > 0.05) relationship in the miscarriage rate with EMT in both groups (**Table 7**).

**Table 7:** Threshold effect analysis of endometrial thickness and clinical outcomes.

Clinical outcomes		Threshold of EMT	OR	95% CI	P
GnRH-a protocol	Clinical pregnancy rate	NA	1.144	(1.081, 1.210)	<0.05
	Live birth rate	NA	1.115	(1.056, 1.176)	<0.05
	Miscarriage	NA	0.996	(0.905,1.095)	>0.05
GnRH-ant protocol	Clinical pregnancy rate	<12mm	1.215	(1.116, 1.324)	<0.05
		≥12mm	0.825	(0.691, 0.985)	<0.05
	Live birth rate	<12mm	1.196	(1.029, 1.163)	<0.05
		≥12mm	0.840	(0.706, 0.999)	<0.05
	Miscarriage	NA	0.955	(0.864, 1.056)	>0.05

GnRH-a, gonadotropin-releasing hormone agonist; GnRH-ant, gonadotropin-releasing hormone antagonist; OR, odds ratio; CI, confidence interval; EMT, Endometrial thickness.

## Discussion

Although there was no significant difference in overall clinical pregnancy outcomes between the two different protocols, the results of curve fitting and threshold effect analyses showed that the clinical pregnancy outcome of the GnRH-a long protocol was significantly better than that of the GnRH-ant protocol when the endometrial thickness exceeded 14 mm. The advantage became more obvious as the endometrium thickened.

Studies (10, 11) have indicated that the great clinical efficacy of GnRH-ant has enabled it to become an effective alternative to GnRH-a. However, the trend of reduced embryo implantation rates observed in some studies (3) has led clinicians to suggest various explanations, and a possible mechanism is reduced endometrial receptivity. Mirkin et al. (12) believed that the GnRH-a long protocol and the GnRH-ant protocol had only slightly affected the endometrial receptivity compared to the natural cycle. In contrast, other studies (13) indicated a stronger effect of the GnRH-ant protocol on the expression of genes related to endometrial receptivity. Still other investigations (14, 15) provided evidence that the GnRH-a long protocol had reduced the endometrial receptivity. Chen et al. (16) found that in the GnRH-ant protocol group, the expression of B-type creatine kinase (CKB), which played an important role in the embryo implantation process, was significantly reduced in the endometrium. The expression of F-actin in the endometrial epithelial cell was reduced, suggesting that actin fiber depolymerization may directly affect the basic function of the cell. The energy metabolism state of the endometrium is also related to the endometrial receptivity. Most functions of endometrial epithelial cells are closely related to the changes in cytoskeleton during the window phase of embryo transfer (17). Cytoskeletal polymerization and depolymerization processes require energy. Chen et al. (18) found that both GnRH-a and GnRH-ant were related to the upregulation of cytoskeletal regulation but down-regulation of energy metabolism, thus negatively affecting endometrial receptivity. Meanwhile, their study also found that complement-mediated immune proteins were only upregulated under GnRH-ant treatment. Immune remodeling of the endometrium is essential for embryo implantation and subsequent placental formation, and immune remodeling disorders can cause reduction of endometrial receptivity or early pregnancy loss (19). That immune destruction was upregulated only under GnRH-ant treatment indicates that GnRH-ant has a stronger negative effect on endometrial receptivity than GnRH-a. On one hand, application of GnRH-ant in the IVF cycle led to an increase in apoptosis-inducing factor-1 (AIF-1) and mediated a large amount of tumor necrosis factor-α (TNF-α) expression during embryo implantation (20), which had an adverse effect on embryo implantation. On the other hand, human uterine natural killer (uNK) cells are the most important lymphocytes in the uterus and play an important role in human pregnancy (21). Infertile women and women with recurrent spontaneous abortion have abnormally elevated NK cell levels (22). Studies (23, 24) had revealed that interference with the uNK cells during embryo implantation may cause implantation failure. The study by Xu et al. (25) demonstrated that the number of uNK cells was significantly higher in the GnRH-ant group than that in the GnRH-a group and the control group during embryo implantation progress. These studies explained the phenomenon that the GnRH-a long protocol was more advantageous in patients with thicker endometrial thickness. However, it remains unknown why the GnRH-ant protocol has the advantage in thinner endometrium.

The advantage of this study lies in the findings of the endometrial threshold in GnRH-a long protocol and GnRH-ant protocol and quantitative relationship between the effect of EMT on clinical pregnancy rate and live birth rate. Some limitations existed including the retrospective study design, single center study, and Chinese patients enrolled only, which may all affect the publication bias. Although strict inclusion and exclusion criteria had been established and confounding factors had been adjusted to control bias, human errors may still exist in the measurement of endometrial thickness due to varied experiences of doctors performing ultrasound examinations. In addition, the number of patients with thin endometrium was insufficient, which has something to do with the fact that thin endometrium is not suitable for embryo transfer. The choice of embryo transfer for patients with thin endometrium was related to their age and embryo status in this study, and if the patient insisted, embryo transfer would be performed after the possibility of endometrial disease and intrauterine adhesions was eliminated. More data are still needed to verify the conclusion of this study even if some significant differences had been achieved in the clinical pregnancy rate and live birth rate between the GnRH-ant and the GnRH-a long protocols.

In summary, the GnRH-ant and GnRH-a long protocols have comparable clinical outcomes in the clinical pregnancy, live birth, and miscarriage rate after propensity score matching. In the medium endometrial thickness of 7 to 10 mm, the clinical pregnancy rate (61.81 *vs* 55.58%) and miscarriage rate (19.43 *vs* 12.83%) in the GnRH-ant regime was significantly higher than those in the GnRH-a long protocol. Subgroup patients with the thickest endometrium had a significantly (P < 0.05) higher clinical pregnancy rate and live birth rate than those in the other subgroups. EMT was an important predictor of clinical outcomes. In the GnRH-a group, the clinical pregnancy rate increased by 14.4% and the live birth rate increased by 11.5% with every 1 mm increase in EMT. In the GnRH-ant group, the EMT threshold for clinical pregnancy rate was 12 mm. When the EMT was thicker than 12 mm, the clinical pregnancy rate decreased by 17.5% for every 1 mm increase in EMT and the live birth rate decreased by 16% with every 1 mm increase in EMT, which indicates that in this range of EMT, the GnRH-ant protocol may have aggravated negative effects on clinical pregnancy as the thickness of the endometrium increases. The GnRH-ant protocol has certain advantages when the EMT is less than 14mm, but the GnRH-a long program has significant advantages after the EMT is thicker than 14mm. Because the EMT can be used as a good indicator for clinical pregnancy outcomes in different protocols, it is recommended to routinely measure the EMT during treatment.

## Data Availability Statement

The raw data supporting the conclusions of this article will be made available by the authors without undue reservation.

## Ethics Statement

The studies involving human participants were reviewed and approved by the Ethics Committee of The Second Hospital of Hebei Medical University. The patients/participants provided their written informed consent to participate in this study.

## Author Contributions

Study design: Z-MZ and G-MH. Data collection: Y-FS, JZ, Y-MX, YH. Data analysis: Y-FS, JZ, and B-JS. Supervision: Z-YL. Writing the original article: Y-FS. Revision: B-LG. All authors contributed to the article and approved the submitted version.

## Funding

People’s Livelihood Science and Technology Project of Hebei Province (20377714D), Natural Science Foundation of Hebei Province in 2019 (H2019206712), Special project of Hebei Provincial Clinical Medicine Research Center (20577710D).

## Conflict of Interest

The authors declare that the research was conducted in the absence of any commercial or financial relationships that could be construed as a potential conflict of interest.
